# A decline in self-esteem in adults over 50 is not found in Japan: age differences in self-esteem from young adulthood to old age

**DOI:** 10.1186/s13104-019-4289-x

**Published:** 2019-05-15

**Authors:** Yuji Ogihara

**Affiliations:** 1 Department of Cognitive Psychology in Education, Graduate School of Education, Kyoto University, Kyoto, Japan; 2 Present Address: Faculty of Science Division II, Tokyo University of Science, Kagurazaka, Shinjuku-ku, Tokyo, 162-8601 Japan

**Keywords:** Self-esteem, Self-liking, Age difference, Development, Culture

## Abstract

**Objective:**

The current research examined age differences in self-esteem in Japan from young adults aged 20 to the elderly aged 69 with a focus on self-esteem trajectories from middle age to old age. Previous research in European American cultures has indicated that self-esteem rises from young adulthood into middle age, and sharply declines starting in one’s 50s or 60s. However, it was unclear whether this sharp drop would be found in Japan. Therefore, the present research investigated whether the same age differences were present in Japan by analyzing data from a large and diverse sample.

**Results:**

Results showed that self-esteem increases from young adulthood into middle age, consistent with previous research. However, the sharp decline after the age of 50 was not found, which is different from the pattern in European American cultures. This may be related to a finding that people in East Asian cultures show more humble attitudes toward themselves.

## Introduction

The average level of self-esteem changes across the life span. However, it is unclear whether this developmental trajectory of self-esteem is found consistently across cultures. The present research examined age differences in self-esteem in Japan, with a focus on self-esteem trajectories between the ages of 50 to 69.

The developmental trajectory of self-esteem in European American cultures has been examined in many studies (especially in the US; for reviews, see [1, 2]). One cross-sectional study that examined age differences in self-esteem across a broad population range (nine to 90 years old) in the US found evidence that the average level of self-esteem is high in childhood, decreases in adolescence, increases in adulthood and peaks at around mid-60s, after which it decreases again [3]. Further evidence for this pattern was observed in a longitudinal study investigating the developmental pattern of self-esteem in the US population from the age of 25 to 104 [4]. Their study demonstrated that self-esteem increases from adulthood to middle age, but begins to decline around the age of 60. These developmental trajectories were also found in another longitudinal study in the US, which showed that self-esteem increases from adolescents to middle adults, reaches a peak at around the age of 50 and decreases among the elderly [5]. This developmental trajectory of self-esteem has also been reported in Germany [6].

Research has indicated that self-esteem peaks in one’s 50s or 60s and then sharply decreases in old age. Two explanations for this drop have been proposed (e.g., [2, 3]). The first is losing things that are important to self-esteem. For example, the elderly lose socioeconomic positions or social roles due to retirement, close others such as spouses and romantic partners, and abilities such as physical and cognitive capacities. The second is a change in attitudes toward oneself. The elderly come to accept their faults and limitations as they get older. This leads them to have more modest, humble and balanced view of themselves.

Prior research has indicated that self-esteem is significantly influenced by culture (e.g., [7, 8]). Hence, the developmental pattern of self-esteem may vary across cultures.

Ogihara investigated age differences in self-esteem from elementary school students to the elderly in Japan [9]. He showed that consistent with the findings obtained in European American cultures [1, 2], self-esteem was high for elementary school students, declined among middle and high school students, and increased from young adults to the elderly. Further, this developmental pattern was also reported in another study that used a separate dataset to examine age differences in self-esteem from middle school students to the elderly in their 60s [10].

However, the developmental trajectory of older adults in Japan remains unclear. Studies in European American cultures have demonstrated that self-esteem decreases sharply from around 50. Although previous research in Japan investigated age differences in self-esteem [9, 10], it compared average scores of self-esteem among aggregated age groups (i.e., 20s, 30s, 40s, 50s and 60s). This analysis could have overlooked a drop in self-esteem after the age of 50. For example, even if the average level of self-esteem of the 60s age group was higher than that of the 50s age group, the average level of self-esteem could have peaked in the late 50s and continued to decrease in the 60s. To reveal the overall developmental trajectory of self-esteem in Japan, it is necessary to examine whether a sharp drop in self-esteem is also found among older adults in Japan in more detail.

Research has demonstrated that people in Japan show more humble, balanced and modest attitudes relative to people in European American cultures (e.g., [7, 11]). Considering that a rise in such humble and balanced attitudes may cause the clear drop in self-esteem in European American cultures, it is predicted that a decline may be less sharp or even absent in Japanese older adults. In other words, there may be a cultural difference in the developmental trajectory of self-esteem.

This research aimed to investigate age differences in self-esteem in Japan, particularly in the older adults over the age of 50. To this end, data from a large and diverse sample in Japan were analyzed.

This data was used in Ogihara [10], but the main purpose of Ogihara [10] was to investigate whether the developmental pattern of self-esteem reported in Ogihara [9] was also found in another independent dataset. Thus, as in Ogihara [9], it compared the average scores of self-esteem in seven aggregated age groups (i.e., middle school students, high school students, 20s, 30s, 40s, 50s, 60s), but did not examine sequential age differences. This analysis may have overlooked a drop in self-esteem around the age of 50. Therefore, the current research examined age differences in self-esteem in more detail by analyzing sequential age differences in self-esteem.

## Main text

### Method

#### Data

The data collected by the National Institute for Youth Education (NIYE; [12]) in 2012 were analyzed. The raw data were obtained by registering with the NIYE.

#### Respondents

Adults aged 20 to 69 from all over Japan participated in a web-based questionnaire study. Sample sizes by gender and generation are shown in Table 1. The total sample was comprised of 2623 males and 2635 females.

**Table 1:** Sample sizes by gender and generation

	20s	30s	40s	50s	60s	Total
Male	522	527	521	528	525	2623
Female	527	529	530	525	524	2635
Total	1049	1056	1051	1053	1049	5258

#### Question items

##### Demographic questions

Participants provided their gender and age.

##### Self-esteem

Participants indicated to what extent the sentence “I like myself” applied to themselves on a four-point scale (1: does not apply at all, 2: does not apply much, 3: applies somewhat, 4: applies very much). Self-liking is one important aspect of self-esteem (e.g., [13, 14]). For ease of interpretation, 1 was subtracted from the scores (i.e., 0: does not apply at all, 1: does not apply much, 2: applies somewhat, 3: applies very much).

#### Analysis

A hierarchical regression analysis was conducted. In Step 1, age was the only independent variable to predict self-esteem. In Step 2, the term age-squared was added as an independent variable. Sample sizes were weighted to give a higher relative weight to data from a large sample.

### Results

A summary of the results is shown in Table 2.

**Table 2:** Summary of regression models in which age predicted self-esteem

		Step 1					Step 2				
Gender		*B*	*SE*	95% *CI*	β	*p*	*B*	*SE*	95% *CI*	β	*p*
Male	Age	0.009	0.001	[.007, .012]	.776	***	0.009	0.001	[.007, .012]	.776	***
	Age^2^						0.000	0.000	[− .000, .000]	− .024	.792
	Δ*R*^2^									.001	.792
	*R* ^2^				.603	***				.603	***
Female	Age	0.010	0.001	[.007, .012]	.772	***	0.010	0.001	[.007, .012]	.772	***
	Age^2^						0.000	0.000	[− .000, .000]	.085	.358
	Δ*R*^2^									.007	.358
	*R* ^2^				.596	***				.603	***

*SE* standard error, *CI* confidence interval

*** p < .001. Independent variables were centered

#### Male

**Fig. 1:**
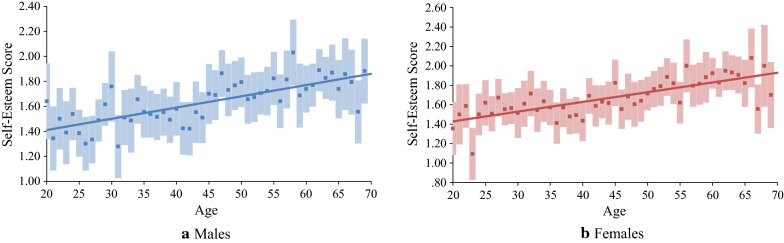
Age differences in self-esteem in Japan. Error bars represent 95% confidence intervals

The average scores of self-esteem and estimated developmental trajectory for males are shown in Fig. 1a. Age significantly predicted self-esteem. However, the term age-squared did not increase the *R*^2^ value significantly (Table 2). Thus, results demonstrated that the average levels of self-esteem continued to increase from 20s to 60s and did not show any decline.

#### Female

The average scores of self-esteem and estimated developmental trajectory for females are indicated in Fig. 1b. Age significantly predicted self-esteem. However, the term age-squared did not increase the *R*^2^ value significantly (Table 2). Thus, results showed that the average levels of self-esteem continued to rise from 20s to 60s without showing any decline.

### Discussion

The present research examined age differences in self-esteem from young adults aged 20 to the elderly aged 69 in Japan. Of particular interest was the pattern of self-esteem among adults over the age of 50. Previous research has indicated that self-esteem continues to increase from young adulthood into middle age, and sharply declines around one’s 50s or 60s in European American cultures (e.g., [3–6]). However, it was unclear whether the same pattern would be found in East Asian cultures. Self-esteem is remarkably influenced by cultural factors (e.g., [7, 8]), so it is possible that a different developmental trajectory of self-esteem is found in East Asian cultures.

Results indicated that self-esteem increases from young adulthood into middle age, which is consistent with previous research (e.g., [3–6]). However, this research did not find evidence of the drop in self-esteem after the age of 50 that has been found in European American cultures. Among both males and females, self-esteem continued to increase from the age of 20 to 69. Therefore, the present research implies that different developmental trajectories are found in different cultural environments. The drop in self-esteem observed in older Western adults is thought to be related to developmental changes: people become more modest and humble in old age (e.g., [2, 3]). In contrast, past research has demonstrated that people in Japan show humble and balanced attitudes, not just in old age (e.g., [7, 11]). This cultural difference may explain the absence of a sharp drop in self-esteem among the Japanese people over the age of 50.

The lack of a clear drop in self-esteem could be interpreted as due to participants reporting their answers on the web: elderly people who use the internet in daily life may be wealthier and healthier than the general elderly, which may obscure a decrease in self-esteem. However, previous research which reported a clear drop in self-esteem among adults in their 60s also used data that was collected online (e.g., [3]). Thus, this explanation seems insufficient to account for the cultural difference in the developmental pattern of self-esteem among people in old age.

## Limitations

This research analyzed age differences in self-liking, which is an important aspect of self-esteem (e.g., [13, 14]). However, it is important to investigate the other aspect of self-esteem (i.e., self-competence). Prior research has indicated that these two aspects were strongly positively related to each other (e.g., [14]). Thus, it is predicted that a consistent developmental pattern would be found in self-competence.

Although this research examined age differences in self-esteem among a diverse sample aged from 20 to 69, and found that a clear drop in self-esteem was absent up to the age of 69, the developmental trajectory after 70 remains unclear. It is possible that a decline occurs in Japan as well but is found later in old age than in previous research (e.g., [3–6]). It is also possible that there is no elderly drop in self-esteem in Japan. To capture the whole picture of the developmental pattern of self-esteem in Japan, it is necessary to investigate self-esteem in the elderly over the age of 70.

This study examined age differences in self-esteem by analyzing cross-sectional data, helping to capture the developmental trajectory of self-esteem in Japan. Yet, cross-sectional data also includes cohort differences. Thus, it is necessary to analyze longitudinal data, which can distinguish between age differences and cohort differences.

## Data Availability

The data that support the findings of this study are available from the National Institute for Youth Education but restrictions apply to the availability of these data, which were used under license for the current study, and so are not publicly available.

## Abbreviations

NIYE: National Institute for Youth Education
SE: standard error
CI: confidence interval
